# High seropositivity of Mumps virus IgG antibodies in unvaccinated population of Mwanza, Tanzania: a community-based study

**DOI:** 10.4314/ahs.v25i1.12

**Published:** 2025-03

**Authors:** Mariam M Mirambo, Helmut Nyawale, Angela B Kalatwa, Bertrand Msemwa, Stephen E Mshana

**Affiliations:** 1 Department of Microbiology and Immunology, Weill Bugando School of Medicine, Catholic University of health and allied sciences, P.O. Box 1464 Mwanza Tanzania; 2 Department of Biochemistry and Molecular Biology, Weill Bugando School of Medicine, Catholic University of health and allied sciences, P.O. Box 1464 Mwanza Tanzania

**Keywords:** Mumps, Unvaccinated population, Mwanza, Tanzania

## Abstract

**Background:**

Mumps virus (MuV) infection has been associated with significant morbidities across the globe. Despite being endemic in most of low- and middle-income countries including Tanzania, it has not been included in routine immunization in many sub-Saharan countries. This study reports MuV IgG seropositivity in different communities of Mwanza, the information that might be useful in devising evidence-based control measures.

**Methodology:**

A laboratory based cross-sectional study was conducted from July to August 2022, involving 276 archived plasma collected from Misungwi, Ukerewe and Magu districts. Socio-demographic information and other relevant information were extracted from database. Indirect Enzyme-Linked Immunosorbent Assay (ELISA) was used to detect MuV IgG antibodies. IBM SPSS version 23 was used for data analysis.

**Results:**

The median age of the participants was 36(Interquartile range (IQR):26-42) years. The overall seropositivity of MuV IgG antibodies was found to be 262 out of 276 (94.93%, (95% Cl:91.59-96.98), highest among age group between 15-24. None of the factors was found to be associated with MuVIgG seropositivity in Mwanza.

**Conclusion:**

MuV IgG seropositivity is alarmingly high in Mwanza suggesting the virus is endemic and might be associated with morbidities. This calls for the need to scale up the serological studies so as to provide evidence for intervention.

## Introduction

Mumps virus (MuV) is an enveloped Ribonucleic Acid (RNA) virus that belongs to the genus Rubulavirus in the family Paramyxoviridae^1^. The viral enveloped particle contains a non-segmented negative strand RNA molecule of 15,384 nucleotides^2^.

Mumps is an acute viral infection that is endemic worldwide affecting mostly children and adolescents^3^. The virus is transmitted through respiratory or oral route with infected respiratory droplets or secretions such as saliva or mucus. The incubation time ranges from two to four weeks^3^. The virus can also spread systemically resulting into viremia in early phase of infection.

Previous reports documented that prior to the introduction of vaccines, 50% of children aged 4-6 years were seropositive while 90% of adolescents years were seropositive^4^,^5^ with sharp increase in mumps antibodies at the age of 3 years^6^. In absence of vaccination the incidence of mumps infection has been found to range from 100-1000 cases/100000 population with epidemic peaks every two to five years^3^,^7^. Worldwide, more than 560,000 cases of mumps infection has been reported between 2005 and 2010^3^.

Risk factors for MuV infections include being unvaccinated and living in crowded conditions (boarding schools, prisons, refugee camps and orphan's houses)^7^. The most notable clinical manifestation of MuV infections is a tender swelling parotid gland which is often self-limited. However, meningitis, abortions and orchitis have been frequently reported in post pubertal individuals with increase in severity as the age increase^2^.

Mumps is vaccine-preventable, and one dose of mumps vaccine is about 80% effective against the disease. Routine vaccination using the combined measles-mumps-rubella has been very effective in reducing MuV infections and associated morbidities in different countries that are implementing MuV vaccine^7^. In Tanzania MuV seropositivity among children has been found to range from 16.7% to 88%^8^-^10^ with recent data from Mwanza reporting seropositivity of about 50% among women with spontaneous abortions^9^-^11^. Introduction of vaccine in a routine immunization programme requires evidence based policy formulation. This study provides data on the magnitude of the MuV infections in different Mwanza communities. This information might be useful in future evidence based control interventions.

## Materials and methods

Study Design, study duration, study area, study population and sample size A cross-sectional laboratory-based study was conducted from July to August 2022 using achieved plasma samples collected from different age groups in the previous community survey in Mwanza, Tanzania. The samples used in this study were collected between July and August 2021, in 3 out of the 8 district councils of the Mwanza region, Tanzania. The selection of the districts was based on the geography of the region with the aim to include both Mwanza, mainland and island. The two mainland districts were Magu and Misungwi, and the island district was Ukerewe. The population of Magu, Misungwi and Ukerewe were 421,119, 467,867 and 387,815 respectively (National Censor 2022) Table 1.

**Table 1:** Population distribution in different age groups in different districts in Mwanza, Tanzania (National Censor 2022)

District		Age groups(Years)		
	0-14	15-64	65 and above	Total
Ukerewe	176,948	195,728	15,136	387,812
Magu	196,183	210,636	14,300	421,119
Misungwi	225,337	227,210	15,320	467,867

The sample size was estimated by Cochran's formulae for finite population and simple random sampling was used to estimate the sample size from the data base provided.

### Data collection and laboratory procedures

Data was extracted from the existing database available at CUHAS Microbiology laboratory. Plasma samples with insufficient volume and incomplete information were excluded. Plasma were taken from -80°c refrigerator at the CUHAS Microbiology laboratory and analyzed for the presence of specific IgG antibodies of mumps by an indirect enzyme linked immunosorbent assay (ELISA) following manufacturer's instructions (Vircell, S.L. Parque Technologico de la Salud, Avicena 8, Spain). The interaction between antigen and antibody resulted into a color formation, the color formed was measured by using a spectrophotometer at 450/620 to measure the optical density. The sample was regarded as positive for mumps antibodies, if optical density was equal or above the cutoff value (OD >1.1).

### Data management and analysis

Laboratory results were recorded into laboratory book and entered into Microsoft excel sheet for cleaning and coding. Data analysis was done by using IBM SPSS Statistics version 23. Percentage and fraction was used to summarize categorical variables including, sex, and residence while median (interquartile range [IQR]) was used for continuous variables including age. Bivariate logistic regression analysis was used to show factors associated with the seropositivity; odds ratio and 95% confidence intervals were determined; and the variables with P-value of less than 0.05 were considered to have statistically significant.

## Results

### Sociodemographic Characteristics

A total of 276 archived plasma samples were included in this study with the median [IQR] age of 36[26-42] years. More than a half 144 (52.8%) were from Misungwi district while most of the samples 168(60.0%) were from female. More than a half of the samples 154(55.8%) were from farmers and the median number of households was 2[IQR:1-4] members (Table 2).

**Table 2:** Sociodemographic characteristics of the enrolled samples in Mwanza (n=276)

Variable		Frequencies(N)	Percentages (%)
Median [IQR] age in years		36[26-42]	
Sex	Male	108	40
	Female	168	60
Age classification(years	0-14	1	0.4
	15-24	48	17.3
	25-59	208	75.4
	60-99	19	6.9
Median [IQR] number in a household		2[1-4]	
District	Ukerewe	41	14.9
	Misungwi	144	52.2
	Magu	91	33.0
Occupation	Businessman	63	22.8
	Employed	20	7.2
	Not working	28	10.1
	Farmer	154	55.8
	*Others	11	4.0

### Clinical Characteristics

Of 276 samples, 101(36.6%), 99(35.9%) and 96(34.8%) reported to have previous history of headache, fatigue, and fever respectively. In addition, 10(3.6%) had diabetes and 36(13%) reported history of sore throat (Table 3).

**Table 3:** Clinical Characteristics of the participants

Variables		Frequencies	Percentages (%)
**History of fever**	**Yes**	96	34.8
	**No**	180	65.2
**History of fatigue**	**Yes**	99	35.9
	**No**	177	64.1
**History of headache**	**Yes**	101	36.6
	**No**	175	63.4
**History of sore throat**	**Yes**	36	13.0
	**No**	240	87.0
**History of diabetes**	**Yes**	10	3.6
	**No**	266	96.4

### Seropositivity of Mumps Antibodies

The seropositivity of mumps IgG antibodies was found to be 262 out of 276, 94.93%,(95% Cl:91.59-96.98). Across the age groups, seropositivity was 100% among the age groups, 0-14 and 15-19 years, and lowest, 94.7%, among 25-59 and 60-99 years age groups (Table 4).

**Table 4:** Distribution of Mumps virus seropositivity in the age groups

Age group	Total number	Seropositive	Percentage (%)
0-14	1	1	100
15-24	48	46	95.8
25-59	208	197	94.71
60-99	19	18	94.7

### Factors associated with seropositivity

None of the factors studied (age, sex, number of people living in a household, history fever, history of sore throat, history of headache and history of diabetes) was found to be associated with Mumps IgG seropositivity on bivariate logistic regression analysis (p>0.2).

## Discussion

This study reports high seropositivity of mumps IgG antibodies among the population in the communities in three districts of Mwanza region. These findings indicates extensive prior exposure to the MuV suggesting that the virus is endemic in Mwanza and might be associated with morbidities. These findings are comparable to other studies in un-vaccinated population in Netherlands and Denmark which reported seropositivity of 95% and 90% respectively^13^, ^14^. However, these findings are also similar to other serological studies in other countries in sub-Saharan Africa among unvaccinated populations with different age structures; a study done in Khartoum, Sudan reported seroprevalence of 97.1% in the age group of 21 years and above^15^. The contrary findings were reported in the Northern part of Africa, Libya, which reported the seropositivity of 43.4% among children ranging from 7-20 years, this is lower compared to the findings in this study which documented higher seroprevalence^16^. The notable discrepancies could be contributed by various factors such as climates, sea-sonality or geographical variations. The current study used achieved plasma that were collected from July to August 2021. Previous studies have reported that in tropical climates transmission tends to be high at any time of the year as compared to other regions^17^-^19^. Further studies to establish seropositivity in different seasons is warranted in our setting. The seropositivity of viral infections tend to increase with increase in age. None of the factors was found to be associated with Mumps IgG seropositivity in Mwanza which could be explained by high seropositivity observed.

## Conclusion

Mumps IgG seropositivity is alarmingly high in Mwanza communities which suggest the virus is endemic in this area. Further studies to establish the clinical significance of MuV infections in our setting are warranted. The data might be useful to devise appropriate evidence based control interventions across the country.

## Data Availability

All data generated/analyzed during this study are included in this manuscript.
